# Evaluation of Cerebral Perfusion Pressure, Cerebral Blood Flow, and Cerebral Oxygenation at Different Head of Bed Positions Using Transcranial Doppler and Near-Infrared Spectroscopy in Postoperative Neurosurgical Patients

**DOI:** 10.7759/cureus.51923

**Published:** 2024-01-09

**Authors:** Nisha Baskar, Manikandan Sethuraman, Ranganatha Praveen, Ajay P Hrishi, Smita Vimala, Unnikrishnan Prathapadas, Mathew Abraham

**Affiliations:** 1 Department of Anesthesiology, Apollo Speciality Hospitals, Madurai, IND; 2 Department of Anesthesiology, Neuroanesthesia Division, Sree Chitra Tirunal Institute for Medical Sciences and Technology, Trivandrum, IND; 3 Department of Neurosurgery, Lisie Hospital, Ernakulam, IND

**Keywords:** postoperative neurosurgical patients, estimated cerebral perfusion pressure, near infrared spectroscopy, transcranial color doppler, head of bed position

## Abstract

Objectives: Nursing postoperative neurosurgical patients with head of bed (HOB) elevation beyond 30° might be desired at times to prevent pulmonary complications. Due to the paucity of studies determining the effect of HOB beyond 30° on cerebral perfusion pressure (CPP), cerebral blood flow (CBF), and regional cerebral oxygenation (rSO2), this study was designed.

Methods: A total of 40 patients following elective neurosurgery for supratentorial tumors were studied in the neurosurgical intensive care unit three hours following admission. They were assessed for CBF velocities of middle cerebral arteries on either side using transcranial color Doppler (TCCD), rSO2 using near-infrared spectroscopy (NIRS), and mean arterial pressure measured at tragus level at various HOB positions. The estimated cerebral perfusion pressure (CPPe) was calculated from TCCD parameters, and the estimated intracranial pressure (ICPe) was then derived. Their variations at different HOB positions were noted.

Results: TCCD parameters such as peak systolic velocity (PSV) and mean flow velocity (MFV) did not significantly vary upon elevating HOB from 0° to 30° but reduced significantly when HOB was further elevated to 60° (p < 0.05). ICPe reduced significantly with a change of HOB positions from 0° to 60° (p < 0.001), and a significant reduction in CPPe was noticed when HOB was elevated to 60° (67.2 ± 10.1 mmHg vs. 74.7 ± 11.2 mmHg at 0°). However, none of these HOB positions affected rSO2 values.

Conclusion: Postoperative nursing with positions up to 60° HOB can be tried in indicated patients following elective neurosurgery when complemented with CBF velocity and rSO2 monitoring and in whom CPP-guided therapy is not preferred.

## Introduction

Nursing patients with the head of the bed (HOB) elevation in intensive care units (ICUs) has been shown to improve oxygenation and minimize the incidence of ventilator-associated pneumonia (VAP) [1,2]. Normal individuals maintain the systemic arterial pressure upon elevating the head from the neutral position by homeostatic reflexes. Elevating the head above the heart is found to cause a reduction in cerebral perfusion pressure (CPP). However, the accompanying reduction in intracranial pressure (ICP) secondary to improved venous drainage would result in a minimal change in cerebral blood flow (CBF) when autoregulation of the cerebral vasculature is intact [3]. Elevating the HOB of patients in whom the autoregulation is impaired can result in the reduction of CBF [3,4]. Current positioning practices in neurocritical care units advocate 30° HOB elevation, extrapolated from traumatic brain injury (TBI) studies, which improved ICP while maintaining the CPP [5].

However, HOB positions beyond 30° might still be desired to prevent postoperative basal atelectasis and pneumonia in neurosurgical patients, especially with a higher body mass index and those who are easily prone to hypoxia in post-endoscopic transnasal transsphenoidal pituitary tumor surgery in acromegalics/Cushing’s in whom continuous positive airway pressure (CPAP) is contraindicated due to fear of tension pneumocephalus [2]. The possibility of secondary brain injury upon reduction in CBF while elevating HOB beyond 30° during the postoperative period can be a concern for them [4]. However, minimal studies are available to date comparing the cerebral hemodynamics between supine and 60° HOB positions [6]. Also, there is a lack of studies regarding postural influences on cerebral hemodynamics in post-neurosurgery patients with supratentorial brain tumors [7]. Non-invasive methods could determine the possibility of secondary brain insults upon reduction in CPP during HOB elevation by assessment of cerebral hemodynamic variations using transcranial Doppler (TCD) [8] and regional cerebral oxygenation (rSO2) using near-infrared spectroscopy (NIRS) as described during shoulder surgery in beach chair position [9]. However, no studies are available to date regarding the use of NIRS as a surrogate marker of cerebral oxygenation at different HOB positions in postoperative neurosurgical patients.

Hence, we designed this study to evaluate CBF velocity, estimated CPP (CPPe), and rSO2 in different HOB positions using transcranial color Doppler (TCCD) and NIRS in neurosurgical patients who underwent surgery for supratentorial tumors.

This study was previously presented as a poster at the 2019 ISNACC Annual Conference of the Indian Society of Neuroanesthesiology and Critical Care (February 15-17, 2019).

## Materials and methods

Our study is a prospective observational study conducted in the neurosurgical ICU of Sree Chitra Tirunal Institute for Medical Sciences and Technology (SCTIMST) on postoperative patients who underwent elective neurosurgery for supratentorial brain tumors, with the study duration of one year. After obtaining the Institutional Ethics Committee approval (SCT/IEC/1259), the study was registered on the Clinical Trials Registry of India (CTRI/2018/11/016291). Written informed consent was obtained, and patients between the ages of 18 and 65 years of either sex, with Glasgow Coma Scale (GCS) score of 14 to 15, American Society of Anesthesiologists (ASA) grade 1 and 2, who underwent elective craniotomy for unilateral supratentorial tumors, with intra-arterial blood pressure monitoring, with hemodynamic stability, and without intraoperative complications were included in our study. Patients with comorbidities such as decompensated heart failure, cardiac shunts, coronary artery disease, valvular disease of the heart, peripheral arterial disease, stroke history, carotid disease, lung diseases, advanced liver or renal disease, bilateral brain pathology, and emergent neurosurgery were excluded from the study.

Recruitment of patients

**Figure 1:**
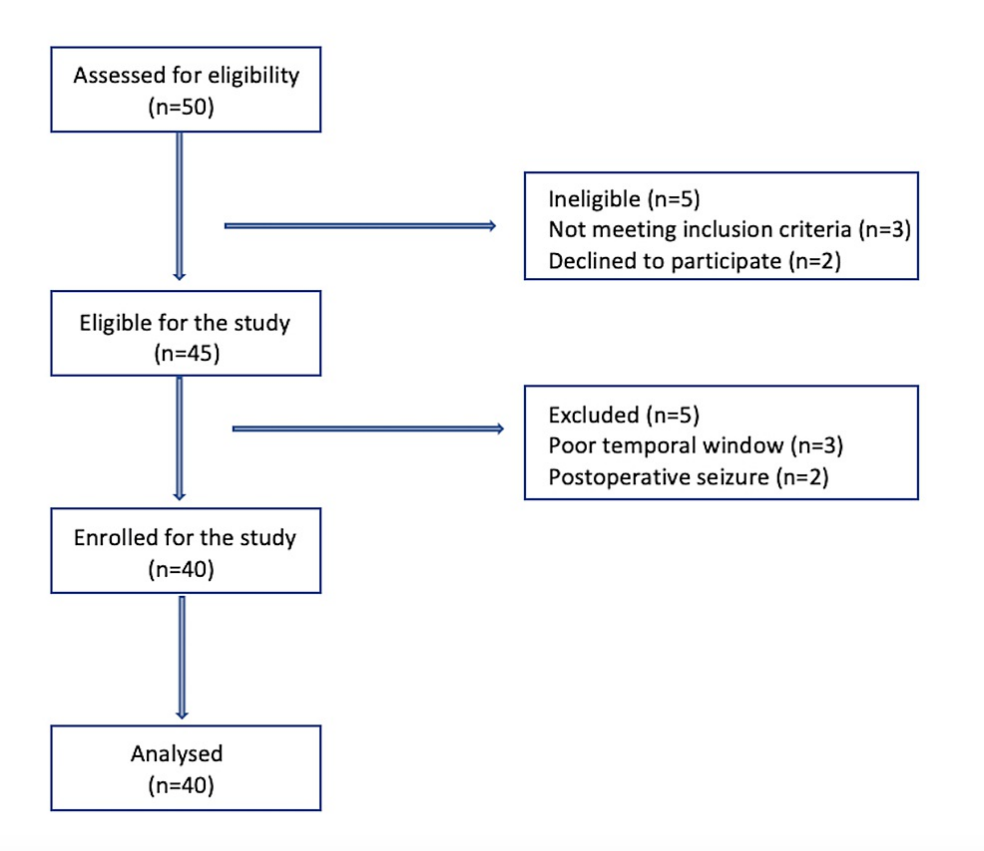
STROBE flowchart: participants screening and enrolment process. STROBE: Strengthening the Reporting of Observational Studies in Epidemiology.

All participants who satisfied the inclusion criteria were included in the study after obtaining written informed consent (Figure 1). Baseline demographic data included age, gender, admission GCS, co-morbidities, and neurological condition. Patients were fasted for eight hours prior to surgery. Tablet pantoprazole (40 mg) and tablet ondansetron (4 mg) were used for premedication. Antiepileptic drugs were continued as per standard protocol.

Intraoperative phase

All patients received general anesthesia as per standard protocol after attaching the ASA standard monitors such as electrocardiogram (ECG), pulse oximeter, and noninvasive blood pressure (NIBP). Following pre-oxygenation, anesthesia was induced with a bolus dose of intravenous (IV) fentanyl (2-3 μg/kg) and IV propofol (1-2 mg/kg) titrated to loss of verbal response and intubated with IV vecuronium (0.1 mg/kg). Anesthesia was maintained with a balanced mixture of air and oxygen, 0.8 to 1 minimum alveolar concentration (MAC) of sevoflurane, atracurium infusion (0.03 mg/kg/hr), and fentanyl infusion (1-2 μg/kg/hr), and ventilated with a tidal volume of 6 to 8 ml/kg body weight to achieve a partial pressure of carbon dioxide (PaCO2) of 35-40 mmHg during surgery. A 20 G radial arterial cannula was placed to monitor real-time blood pressure, which was maintained within 20% of baseline using mephentermine boluses, and a peripherally inserted central line was secured. Intraoperatively fluids were administered to target the urine output of 0.5 to 1 ml/kg/hr while maintaining a pulse pressure variation < 12%. The threshold for blood transfusion was fixed at a hemoglobin value of 8 g/dl. Emergence and extubation were performed as per standard protocol.

Immediate postoperative phase in the ICU

In the ICU, patients were monitored for saturation of peripheral oxygen (SpO2), ECG, and invasive blood pressure; oxygen was administered via face mask and neurologically assessed, including GCS score, pupil size, and reactivity. Arterial blood gases (ABG) were analyzed after one hour of extubation.

Study protocol in the ICU

The study commenced three hours following ICU admission. Upon stabilizing the patients in the supine position for about five minutes with HOB 0°, the arterial transducer was placed at phlebostatic axis level (PS), which is at the intersection of the midpoint of anterior and posterior chest line and fourth intercostal space and zeroed. Baseline values of heart rate (HR), systolic blood pressure (SBP) at PS (SBP-PS), diastolic blood pressure (DBP) at PS (DBP-PS), mean blood pressure (MBP) at PS (MBP-PS), respiratory rate (RR), and SpO2 were recorded. Next, the transducer was shifted to the level of tragus (T) and zeroed. Baseline SBP-T, DBP-T, and MBP-T were noted. Frontal rSO2 values were then noted on either side, i.e., ipsilateral (NIRS-op) and contralateral to surgery (NIRS-nonop). Using a Doppler ultrasound probe (phased array 2 MHz), the middle cerebral artery was insonated at an approximate depth of 45-50 mm to obtain measurements from TCCD. TCCD signal when obtained was allowed to stabilize for a couple of minutes for the generation of cerebral blood flow velocity to time waveforms, whose values would then be displayed on the screen of the monitor. The color indicated the relative direction of flow across the probe. A couple of readings five minutes apart were documented. Details of TCCD insonation were carefully noted as a baseline, thereby facilitating appropriate measurement of these parameters at various HOB positions. The TCCD parameters were recorded on either side, i.e., sides ipsilateral and contralateral to surgery. Baseline SBP (PS and T), DBP (PS and T), MBP (PS and T), peak systolic flow velocity (PSV), mean flow velocity (MFV), end-diastolic velocity (EDV), pulsatility index (PI), and NIRS were noted. MBP here was also the same as mean arterial pressure (MAP). Similar parameters were recorded at HOB 30° and 60° (after five minutes following stabilization in respective positions) and their values were compared with baseline. The HOB of the patient was gradually elevated over 30 seconds from one level to another using a protractor (the protractor was placed vertically at the edge of the bed and angle was measured, a common practice in our ICU). Using the values of the above parameters, CPPe [10] and estimated intracranial pressure (ICPe) were calculated by the following formulas:

CPPe (estimated CPP) = MAP-T × EDV/MFV+14.

ICPe (estimated ICP) = MAP-T - CPPe.

Data collection

Demographics (age, sex, GCS preoperative and postoperative, and tumor characteristics), intraoperative hemodynamic details, ABG, immediate postoperative hemodynamic details (HR, SBP, DBP, MBP), SpO2, and recovery details were recorded. TCD parameters such as PSV, EDV, MFV, and PI at various HOB positions (0°, 30°, and 60°) and rSO2 values at 0°, 30°, and 60° HOB positions were recorded. MAP-PS and MAP-T were recorded at 0°, 30°, and 60° HOB positions.

Sample size calculation and statistical analysis

The sample size for this prospective observational study was calculated based on a previous study by Petersen et al. [11]. Assuming maximum variation in cerebral perfusion pressure at 60° HOB to be 10% as compared to baseline, for an alpha of 5% and a beta of 80%, the sample size was calculated to be 15 (including accounting for dropouts). To compensate for the non-favorable TCCD window in about 10-20% of the overall population, five more additional patients needed to be included. Finally, we recruited 40 patients to improve the validity of our study.

SPSS version 25.0 (IBM Corp., Armonk, NY) was used for the statistical analyses in our study. Percentage, frequency, median (interquartile range), and mean (standard deviation) represented the descriptive data. The test used for the comparison of variations in CBF velocity, TCCD, NIRS, CPPe, and ICPe among various HOB positions was one-way repeated measures variance analysis (ANOVA). Mauchly's sphericity test validated the assumed sphericity. Upon violation of sphericity, Greenhouse-Geisser correction was used to minimize type I error. A p-value of ≤0.05 was considered to be statistically significant. Relative variations in parameters at various HOB positions were determined as percent (%) difference between the various positions as:

% change from HOB 0° to HOB 30° = (HOB 30° - HOB 0°/HOB 0°) x 100.

% change from HOB 30° to HOB 60° = (HOB 60° - HOB 30°/HOB 30°) x 100.

% change from HOB 0° to HOB 60° = (HOB 60° - HOB 0°/HOB 0°) x 100.

An independent sample t-test was used to compare mean CBF velocity and rSO2 values between the operative and non-operative sides of the brain.

## Results

Forty of the 50 patients assessed for eligibility were finally enrolled, as shown in Figure 1. Five patients did not fulfill the inclusion criteria and were hence excluded. Three patients who had a poor trans-temporal window and two other patients who witnessed seizures (generalized tonic-clonic) in the postoperative period were eventually excluded. All the 40 recruited patients successfully completed the study. Of the 40 patients enrolled, 20 were males and 20 were females, whose mean age was 40 (±14) years. Preoperatively, the median GCS score was 15 (15-15), while a median score of 14 (14-15) was seen postoperatively in the ICU. The most common diagnosis among our neurosurgical patients was glioma (55%). No adverse events occurred in any of the patients during elevation of HOB at various time frames (Table 1).

**Table 1:** Patient characteristics. GCS: Glasgow Coma Score.

Parameters	n = 40
Gender (male:female) (numbers)	20:20
Age (years), mean ± SD	40 ± 14
GCS (on admission), median (IQR)	15 (15-15)
GCS (postoperative), median (IQR)	14 (14-15)
Diagnosis (numbers) (percentage)	
Glioma	22 (55%)
Meningioma	6 (15%)
Dysembryoplastic neuroepithelial tumor	3 (7.5%)
Pineal region tumor	3 (7.5%)
Lateral ventricle tumor	3 (7.5%)
Colloid cyst	2 (5%)
Thalamic lesion	1 (2.5%)

Upon elevating HOB from 0°to 30°, there was no significant change in the TCD parameters such as PSV, MFV, and PI at both the operative and non-operative sides (p > 0.05) (Tables 2-4). While no significant change was found in EDV on the non-operative side, the operative side did show a substantial fall in EDV, with a 6.6% drift from baseline with p = 0.008. Elevating the HOB from 30° to 60° showed a significant reduction in TCD parameters such as PSV, EDV, and MFV without any substantial change in PI values at both operative and non-operative sides. There were no significant changes in the NIRS values upon elevating the HOB from 0° to 30° and 30° to 60°, and the calculated difference between 0° to 60° at operative (p = 1.00, p = 1.00, and p = 0.052) and non-operative sides (p = 1.00, p = 1.00, and p = 0.641), respectively (Tables 2-4).

**Table 2:** TCCD-derived cerebral blood flow velocities and NIRS-derived regional cerebral oxygenation (rSO2) values on the operative and non-operative sides. TCCD: transcranial color Doppler; NIRS: near-infrared spectroscopy; PSV: peak systolic velocity; EDV: end-diastolic velocity; MFV: mean flow velocity; PI: pulsatility index; HOB: head of bed.

Operative side						Non-operative side					
HOB	PSV (cm/sec), mean ± SD	EDV (cm/sec), mean ± SD	MFV (cm/sec), mean ± SD	PI, mean ± SD	rSO2 (%), mean ± SD	HOB	PSV (cm/sec), mean ± SD	EDV (cm/sec) mean ± SD	MFV (cm/sec), mean ± SD	PI, mean ± SD	rSO2 (%), mean ± SD
0°	79.9 ± 17.0	34.8 ± 11.1	52.7 ± 13.7	0.88 ± 0.19	71.1 ± 7.7	0°	81.4 ± 19.3	36.1 ± 11.8	54.4 ± 15.2	0.83 ± 0.15	70.4 ± 6.9
30°	79.4 ± 17.5	32.5 ± 9.8	51.3 ± 12.8	0.91 ± 0.17	71.4 ± 8.3	30°	79.9 ± 18.4	34.4 ± 9.9	51.9 ± 13.9	0.89 ± 0.21	70.4 ± 7.0
60°	74.4 ±17.3	30.4 ± 8.8	47.19 ± 11.9	0.93 ± 0.17	70.9 ± 8.1	60°	75.2 ± 16.7	31.7 ± 7.8	48.9 ± 11.4	0.89 ± 0.19	70.2 ± 7.4

**Table 3:** Relative percentage changes and difference of mean in TCCD-derived flow velocities and rSO2 values from NIRS with various head of bed positions on the operative side of the brain. TCCD: transcranial color Doppler; NIRS: near-infrared spectroscopy; MD: mean difference; PSV: peak systolic velocity; EDV: end-diastolic velocity; MFV: mean flow velocity; PI: pulsatility index; HOB: head of bed; rSO2: regional cerebral oxygenation.

HOB (change of position from-to)	PSV		EDV		MFV		PI		rSO2	
	% change (MD)	P-value	% change (MD)	P-value	% change (MD)	P-value	% change (MD)	P-value	% change (MD)	P-value
0°-30°	-0.6 (-0.52)	1.000	-6.6 (-2.26)	0.008	-2.7 (-1.40)	0.690	5.7 (0.02)	0.981	0.4 (0.3)	1.000
30°-60°	-6.3 (-5.08)	<0.001	-6.5 (-2.11)	0.030	-6.5 (-4.2)	<0.001	2.2 (0.02)	0.330	0.3 (0.18)	1.000
0°-60°	-6.9 (-5.59)	0.001	-12.6 (-4.4)	0.001	-10.6 (-5.6)	<0.001	4.5 (0.04)	0.301	-0.7 (-0.5)	0.052

**Table 4:** Relative percentage changes and difference of mean in TCCD-derived flow velocities and rSO2 values from NIRS with various head of bed positions on the non-operative side of the brain. TCCD: transcranial color Doppler; NIRS: near-infrared spectroscopy; MD: mean difference; PSV: peak systolic velocity; EDV: end-diastolic velocity; MFV: mean flow velocity; PI: pulsatility index; HOB: head of bed; rSO2: regional cerebral oxygenation.

HOB (change of position from-to)	PSV		EDV		MFV		PI		rSO2	
	% change (MD)	P-value	% change (MD)	P-value	% change (MD)	P-value	% change (MD)	P-value	% change (MD)	P-value
0°-30°	-1.7 (-1.36)	0.660	-5.1 (-1.7)	0.070	-4.5 (-2.46)	0.120	7 (0.06)	0.350	0.4 (0.3)	1.000
30°-60°	-6.0 (-4.81)	<0.001	-7.4 (-2.68)	0.007	-5.7 (-2.98)	0.007	1.1 (0.01)	0.440	-0.2 (-0.18)	1.000
0°-60°	-7.6 (-6.18)	0.001	-12.1 (-4.4)	0.001	-10.1 (-5.5)	<0.001	6 (-0.05)	0.140	-0.1 (-0.1)	0.614

Upon comparing MFV and rSO2 values between the operative and non-operative sides at various HOB positions, no significant variations were found in MFV and NIRS on the operative side when compared with the non-operative side upon elevating HOB from 0° to 30° and 30° to 60° and calculated difference between 0° and 60° (p > 0.05) (Table 5).

**Table 5:** Comparison of relative percentage variations and difference of mean in MFV and rSO2 values from NIRS on the surgical with the non-surgical side at different HOB positions. NIRS: near-infrared spectroscopy; MD: mean difference; OS: operative side; NOS: non-operative side; MFV: mean flow velocity; HOB: head of bed; rSO2: regional cerebral oxygenation.

HOB (change of position from-to)	MFV % change (MD) - OS	MFV % change (MD) - NOS	P-value	rSO2 % change (MD) - OS	rSO2 % change (MD) - NOS	P-value
0°-30°	-2.7% (-1.40 ± 13.7)	-4.5% (-2.46 ± 8.9)	0.466	0.4% (-0.3 ± 3.4)	0.4% (-0.3 ± 3.3)	0.636
30°-60°	-6.5% (-4.2 ± 12.8)	-5.7% (-2.98 ± 13.9)	0.236	-0.3% (-0.2 ± 3.3)	-0.2% (-0.18 ± 2.3)	0.623
0°-60°	-10.6% (-5.6 ± 11.9)	-10.1% (-5.5 ± 11.4)	0.628	-0.7% (-0.5 ± 3.4)	-0.1% (-0.1 ± 3.9)	0.864

The hemodynamic parameters measured at different HOB positions at 0°, 30°, and 60° did not vary significantly among themselves when the transducer was placed at the phlebostatic axis (at heart level) (p > 0.05). However, there was a significant reduction in SBP, DBP, and MBP among these different positions when the arterial transducer was placed at the level of the tragus (p < 0.01) (Table 6).

**Table 6:** Hemodynamic variations at phlebostatic axis level transducer and tragus level transducer placement with various head of bed positions. SBP: systolic blood pressure; DBP: diastolic blood pressure; MBP: mean blood pressure; HOB: head of bed; PS: phlebostatic axis level; T: tragus level.

HOB	SBP (PS), mmHg (mean ± SD)	DBP (PS), mmHg (mean ± SD)	MBP (PS), mmHg (mean ± SD)	SBP (T), mmHg (mean ± SD)	DBP (T), mmHg (mean ± SD)	MBP (T), mmHg (mean ± SD)
0°	130.7 ± 16.3	73.6 ± 12.1	92.3 ± 13.3	129.8 ± 16.8	73.9 ± 11.4	92.7 ± 13.4
30°	130.0 ± 14.3	73.1 ± 10.8	91.5 ± 12.3	124.9 ± 14.5	68.2 ± 10.8	86.7 ± 12.1
60°	127.6 ± 16.5	71.5 ± 10.4	89.4 ± 12.8	119.6 ± 14.9	64.3 ± 9.8	82.4 ± 11.7
P-value	0.067	0.064	0.061	<0.001	<0.001	<0.001

ICPe and CPPe were calculated using MAP value at the level of tragus. There was a significant reduction in ICPe (p < 0.01) when the HOB was elevated from 0° to 30° and 30° to 60° and did not significantly reduce the CPPe (p > 0.05). However, the calculated difference between 0° and 60° had a significant reduction in CPPe from a mean of 74.7 (baseline) to 67.2 (p < 0.01) (Table 7).

**Table 7:** Relative percentage changes and differences of mean in estimated cerebral perfusion pressure (CPPe) and intracranial pressure (ICPe) with various head of bed positions. MD: mean difference; HOB: head of bed.

HOB	CPPe, mmHg (mean ± SD)	Absolute MD and relative % change (p-value) - 0°-30°, 30°-60°, 0°-60°			ICPe, mmHg (mean ± SD)	Absolute MD and relative % change (p-value) - 0°-30°, 30°-60°, 0°-60°		
0°	74.7 ± 11.2	3.02, 4.04% (0.08)		7.45, 10% (<0.001)	16.4 ± 4.8	2.53, 15.4% (<0.001)		4.58, 28% (<0.001)
30°	71.7 ± 9.5		4.48, 6.2% (0.07)		13.9 ± 4.4		2.05, 14.7% (<0.001)	
60°	67.2 ± 10.1				11.8 ± 4.3			

## Discussion

This study was designed to evaluate the CPP, CBF velocity, and rSO2 at different HOB positions using TCCD and NIRS in neurosurgical patients who underwent surgery for supratentorial tumors. Though we found no significant changes in TCCD parameters (such as PSV, EDV, and MFV) upon elevating the HOB from 0° to 30°, we found a substantial reduction in all these parameters when the HOB was elevated from 30° to 60°. There was a significant reduction in ICPe upon changing these positions from 0° to 60°. Also, we found a significant decrease in CPPe when HOB was elevated to 60°. However, no significant variations in PI (as determined by TCCD) and rSO2 values (as determined by NIRS) were found at any of the positions. Also, there was no significant difference between operative and non-operative sides concerning TCCD or NIRS parameters.

An essential aspect of neuro-intensive care is to ensure optimum CBF and oxygenation for which the patients are nursed in the most ideal HOB position, which would also prevent an increase in ICP [5]. The association of CBF to cerebral tissue metabolism is vital, such that the alterations in CBF can lead to secondary brain injuries in deranged cerebral autoregulation [7]. Dynamic situations in neurosurgical patients, such as surgery for brain tumors, cerebrovascular disorders, and postoperatively nursed head and body positions, may alter CBF and CPP [12]. Results of previous studies regarding the association of CBF velocity and CPP with HOB are twofold, with a decrease in CBF and CPP upon HOB elevation in some studies and without any variation in CBF velocity with HOB elevation from 0° to 30° in others [13-16]. In a study on postoperative brain surgery patients, there was an initial reduction in CBF velocity with HOB elevation up to 45° [17]. TCD findings on subarachnoid hemorrhage patients did not demonstrate any changes in CBF velocity at various HOB positions [18,19]. A study on acute ischemic stroke patients demonstrated variations in CBF velocities at HOB 30° in patients with partially recanalized blood vessels (that were responsible for stroke) as compared to patients with completely recanalized blood vessels, who showed no such variations [20]. Also, there were no changes in CBF velocities at various HOB positions in healthy volunteers [21,22]. There needs to be more literature involving 60° HOB positions and more studies on postoperative patients who underwent elective neurosurgery for brain tumors [7,23]. The majority of the studied population belonged to either TBI, subarachnoid hemorrhage (SAH), or acute stroke patients. Also, none of the previous studies in the neurosurgical population used a combination of noninvasive monitoring of cerebral oxygenation, such as NIRS, and monitoring CBF velocities, such as TCD, at various HOB positions.

Our findings of insignificant change in CBF velocities of the middle cerebral artery when the HOB of the patient was elevated from 0° to 30° were similar to the results of a study on the influence of various HOB positions on CBF velocity [7]. HOB positions of 0° and 30° were assessed and their study population matched ours, who underwent intracranial surgery for tumors. We found a significant reduction in the MAP when HOB was elevated from 0° to 30°, which was not the case in the study by Kose and Hatipoglu, who had no such variations in MAP [7]. They attributed these findings of stable cerebral and systemic hemodynamics to normal cerebral autoregulation and intactness of the body’s compensatory mechanisms. However, it was notable that their TCD examinations were performed after 72 hours of surgery, during which the systemic and cerebral homeostasis would have normalized [7].

We found a significant reduction in CBF velocities (on both the hemispheres) and MAP upon elevation of HOB to 60°. CPPe derived from TCCD parameters was significantly reduced upon elevation of HOB to 60° (10% reduction as compared to baseline) while no significant variations were noted at 30°. There was a significant reduction in ICPe when HOB was elevated to 30° and 60°. This was similar to studies involving comatose patients due to various intracranial conditions, where reduction in CPP and CBF was seen at HOB 45° [12], and in a group of 33 patients with GCS 3-8 upon elevation of HOB from 0° to 60°, where significant improvement in ICP with a reduction in MAP and CPP was noticed [6]. Yang et al. studied TCD measurements in post-cerebral operation patients at 72 hours following surgery. Early reduction in CBF velocities followed by maintenance of CBF was noted at 30° and 45° HOB positions, attributing to the intact autoregulation maintaining CBF [17]. In another study, there was no change in CBF velocity in various HOB positions at 0° to 90° in SAH patients when assessed after 72 hours [15]. A study on severe TBI patients showed ICP reduction without any variations in MAP, CPP, and cerebral oxygenation (assessed by invasive monitors such as jugular venous oxygen saturation (SjvO2) and brain tissue oxygen tension (PbtO2)) at HOB 30° [16]. We made an attempt to ensure CBF adequacy at HOB 30° and 60° by monitoring rSO2 using NIRS, thereby trying to add credibility to the results of our study. Even though the variation in mean CPPe value was significant at HOB 60°, it still remained within the limitations of Brain Trauma Foundation (BTF) recommendations [5]. Also, there was no significant change in the NIRS values even at 60° HOB. Previous studies on NIRS demonstrated cerebral desaturation (>20% reduction in the rsO2 values as compared to baseline) during shoulder surgery in the beach chair position, thereby suggesting its essential role in detecting cerebral hypoperfusion at various HOB positions [9]. Schramm et al. described the beneficial effect of intraoperative sitting position on the betterment of cerebral oxygenation (using NIRS) when cardiac output was constantly maintained during cranial surgeries. They hypothesized that by reducing the ICP, sitting position could improve cerebral oxygenation in intracranial pathologies [24]. Another study found a gradual reduction in rSO2 in sitting and prone positions during posterior fossa surgery without any statistical difference between them. MAP was found to be lower in sitting position as compared to prone [25]. The authors speculated that even though the MAP was maintained >55 mmHg, minimal elevation of the lower limit of MAP would have improved rSO2 values in the sitting position [25].

A retrospective study involving orthopedic surgery in the beach chair position and neurosurgery patients in the sitting position found that MAP obtained from transducers at the level of the heart was significantly higher as compared to transducers at the tragus level during the sitting position. However, clinically, this absolute difference between MAP at the heart and tragus level was found to be small [26]. Our findings were similar in terms of statistically higher MAP-PS value as compared to MAP-T; however, the clinical difference was significantly higher, unlike the above retrospective study. We attribute our findings to the possible positional difference of the legs during the sitting position in the operation theater where legs are placed at heart level while only HOB is elevated in the ICU. Also, debates from previous studies regarding the placement of arterial transducer (PS vs. T level) to obtain the MAP from which CPPe was derived were inconclusive until a recent study by Tse et al. favored slightly toward MAP-T [27,28]. We obtained CPPe using MAP-T values and we felt that the measurement of MAP-T can minimize perioperative ischemia.

Various intraoperative factors such as brain retraction, surgical stress, and trauma secondary to dissection or perioperative cerebral edema could have altered cerebral autoregulation in our study population, which might have contributed to our overall findings. The timing of our assessment, which was three hours following immediate surgery, would have also added to our results, as the compensatory homeostatic mechanisms, which would otherwise regulate systemic and thereby cerebral hemodynamics, would not have completely recovered during this period. However, most studies with alternative results were performed after 72 hours of the postoperative period, during which these homeostatic mechanisms could have recovered.

Limitations

Single-time assessment of physiological parameters was performed immediately in the postoperative period. In view of constant fluctuations in the physiological variables in the neuro-ICU scenario, it can be realized that a single recording might not reflect overall importance, and hence, multiple recordings would be desired. However, we aimed to detect the variations in the cerebral hemodynamics secondary to progressive elevations in the HOB. The measured TCCD velocities as a surrogate for CBF have recognized limitations, including its operator dependency. Also, our measurements focused only on the middle cerebral artery, and the venous pressure (surrogate of downstream pressure), which is postural dependent and could affect CBF, was not measured. This study was conducted on neurosurgical patients who underwent elective surgery for supratentorial tumors; hence, there is a high possibility of all our patients having ICP less than 20 mmHg and also they all had spontaneous respiration. Hence, we acknowledge the need for further studies to validate our findings prior to their implementation on mechanically ventilated patients and in whom ICP values are high.

## Conclusions

Variations in arterial blood pressure and CBF velocities are seen with the elevation in the HOB in postoperative neurosurgical patients without much significance up to HOB 30°, as determined by various studies. Though we found significant reductions in CBF velocities and CPPe upon elevating HOB to 60°, there was no corresponding reduction in regional cerebral oxygen saturation (as determined by NIRS). At the same time, there was an improvement in ICPe. Even the value of CPPe, which was reduced at HOB 60°, was within the safe limits of the BTF recommendations. Nursing the patients at 60° HOB position in the postoperative period may prevent pulmonary complications when applied in the presence of CBF velocity (TCD) and cerebral oxygenation monitors (NIRS). We, however, recommend exercising caution in elevating the HOB to 60° when the CPP-guided management is preferred. Determining the desired HOB position facilitating postoperative lung parameters without compromising the cerebral perfusion may be possible after further validation of bedside rSO2 monitor (NIRS) and transcranial cerebral perfusion monitors at individual levels. In the era of daycare neurosurgery, which focuses primarily on early mobilization, a complete knowledge of cerebral hemodynamics at various HOB positions would facilitate overall management. However, further studies on various HOB positions with larger sample sizes would improve the overall care of these patients.
